# Sensitive and rapid detection of *Culex pipiens* and *Aedes albopictus*

**DOI:** 10.3389/finsc.2023.1015695

**Published:** 2023-03-02

**Authors:** Xiao Wei, Biao Meng, Yan Li, Hong Peng, Xiangna Zhao

**Affiliations:** ^1^ Department of Pest Management, Centers for Disease Control and Prevention of People's Liberation Army (PLA), Beijing, China; ^2^ Department of Epidemiology and Biostatistics, School of Public Health, Anhui Medical University, Hefei, China

**Keywords:** *Culex pipiens*, *Aedes albopictus*, rapid detection, LAMP, mass screening

## Abstract

**Background:**

*Culex pipiens* and *Aedes albopictus* are closely related to human life, and transmit a variety of viruses, causing serious harm to human health. Cytochrome c oxidase I (COI) gene has been selected as a marker gene for studying phylogeny and molecular evolution of species and is also an effective molecular marker for studying the evolutionary mechanism and systematic reconstruction of diptera insects.

**Methods:**

A loop-mediated isothermal amplification (LAMP) method for the rapid and sensitive detection of *Cx. pipiens* and *Ae. albopictus* were first described in this study. The experimental results were verified by real-time PCR.

**Results:**

Our study showed the lower limit of sample concentration that can be detected by LAMP method is 0.5 pg/μl within 20 min for *Cx. pipiens*, and 1 pg/μl within 20 min for *Ae. albopictus*, which were more sensitive than PCR method. Validation tests with field samples showed LAMP method had good specificity and sensitivity and could identify the target species quickly and accurately.

**Conclusion:**

The LAMP method developed in this study allowed the rapid and sensitive detection of *Cx. pipiens* and *Ae. albopictus*, which will be expected to be used for mass screening in batches of the field.

## Background

*Culex pipiens* are cosmopolitan species and can transmit many arboviruses, including Japanese encephalitis virus, West Nile virus, human lymphatic filariasis. Mosquitoes of the *Cx. pipiens* are primary vectors of the West Nile virus in Europe and North America, and the transmission cycle involves birds and mosquitoes (1). *Ae. albopictus* originated from Southeast Asia, and over the past 50 years, they have successfully colonized most of the tropical, subtropical, and temperate regions worldwide (2). Currently, *Ae. albopictus* is the most common and widespread mosquito species in Southeast Asia and China (3). *Ae. albopictus* is a very aggressive biter and can transmit a variety of pathogens, including Dengue virus, Ross River virus, etc.

*Cx. pipiens* and *Ae. albopictus* are closely associated with human life because of human blood feeding habits of the female mosquitoes. Based on the physiological characteristics and specific behavior of these mosquitoes (2). In addition, mosquito bites can cause severe allergies and other discomfort in humans (4). Due to the lack of effective vaccines or drugs for many insect-borne diseases, as well as the rapid evolution of insecticide resistance, many insect vectors are posing a serious threat to human health and the environment (5).

At present, there are many problems in the traditional morphological identification methods of medically important insect vectors, such as the limitations of identifying the different developmental stages and sexes and damaged specimens, the difficulty of accurate identification of closely related species, and the inability of accurate and rapid identification of exotic medical vectors due to the lack of reference materials and specimens.

Cytochrome c oxidase I (COI) gene is one of the three cytochrome oxidase subunits encoded by mitochondrial genes (6), and it is the gene with the largest molecular weight and the most conserved functional domain (7). The COI gene is characterized by strict maternal inheritance, conserved genetic makeup, and moderate evolution rate, few insertions and deletions, and easy amplification by universal primers (6, 8). Therefore, COI has been used as a marker gene for studying phylogeny and molecular evolution of species, including mammals, fish, insects, birds, and other species (6, 7, 9), and is also an effective molecular marker for studying the evolutionary mechanism and systematic reconstruction of Diptera insects (10).

Loop-mediated isothermal amplification (LAMP) is a widely used one-step nucleic acid amplification method suitable for gene diagnosis (11, 12). LAMP has the advantage of high sensitivity and specificity, and the only equipment required is a heating source with a thermostat, which has strong appeal in the field of developing low-cost, real-time detection (12, 13).

The selection of target genes is one of the most important factors in LAMP detection. The COI gene has large sequence differences among different species of mosquitoes, which can be used for specific detection of *Cx. pipiens* and *Ae. albopictus*, and has important potential application value for species identification of a large number of samples or incomplete biological samples.

Effective and rapid surveillance of mosquitoes is critical in many cases, but this becomes increasingly difficult as the number of samples increases. In this study, we established a highly sensitive and rapid LAMP detection method to specifically identify the *Cx. pipiens* and *Ae. albopictus*, respectively.

## Methods

### Mosquitoes

A colony of *Ae. albopictus* was established and maintained in our laboratory. The larvae were fed on rat chow. Adult *Ae. albopictus* were fed on 10% sucrose and reared at 28°C, 80% relative humidity and 16:8 light-dark cycle. *Cx. pipiens* and other mosquito species were acquired from the wild in Beijing, China. The mosquitoes caught in the wild were categorized into *Cx. pipiens*, *Culex tritaeniorhynchus*, *Anopheles sinensis*, *Aedes vexans*, and *Armigeres subalbatus*.

### Preparation of templates

DNA samples were extracted from mosquitoes fed in our laboratory and captured in the wild. Nucleic acid from the legs of mosquitoes was extracted using the TIANamp Genomic DNA Kit. The concentrations of DNA were determined by a Nanodrop Lite spectrophotometer (Thermo Fisher, China).

### Primer design

LAMP primers were designed targeting the COI genes published at NCBI (GenBank LC646367.1 for *Cx. pipiens*, GenBank KX266719.1for *Ae. albopictus*). We designed specific primer sets for the LAMP detection of the *Cx. pipiens* and *Ae. albopictus* by LAMP primer designing software Version 5 (http://primerexplorer.jp/lampv5e/index.html). Each set included an outer forward primer (F3), an outer backward primer (B3), a forward inner primer (FIP), a backward inner primer (BIP), and backward loop primer LB (optional) (**Table 1**). All primers were synthesized commercially (Sangon Biotech Co., Ltd, Shanghai, China).

**Table 1:** Primer sets for the LAMP detection of the *Cx. pipiens* and *Ae. albopictus*, respectively.

Primer Set	Primer	Sequences (5’to3’)
Primer sets for the LAMP detection of *Cx. pipiens* in this study		
C1	C.pip-1-F3	GGATTTGGAAATTGATTAGTTCCT
	C.pip-1-B3	ACTGAAGCTCCAGCATGA
	C.pip-1-FIP	GTGTCAATGAAGGAGGTAGTATTCATTTTTTAGGAGCTCCAGATATGGC
	C.pip-1-BIP	TAGTTTAGTAGAAAATGGAGCTGGGTTTTGCTGTTCCAGATGAAAGAGG
C2	C.pip-2-F3	ACCAGGTGTATTTATTGGAAAT
	C.pip-2-B3	CTCCATTTTCTACTAAACTACTTGA
	C.pip-2-FIP	TCCAAATCCTCCAATTATGATTGGTTTTTGTTATTGTAACTGCTCATGCTT
	C.pip-2-BIP	TTCCTTTAATGTTAGGAGCTCCAGATTTTAGTGTCAATGAAGGAGGTAG
C3	C.pip-3-F3 C.pip-3-B3 C.pip-3-FIP C.pip-3-BIP C.pip-3-LB	TTATTGTAACTGCTCATGCTT CCAGATGAAAGAGGGGGA GCCATATCTGGAGCTCCTAACATTATTTTGTAATACCAATCATAATTGGAGGAT TGAATACTACCTCCTTCATTGACACTTTTACTGTTCATCCAGTCCCA CTTTCAAGTAGTTTAGTAG
Primer sets for the LAMP detection of *Ae. albopictus* in this study		
A1	A.albopi-1- F3 A.albopi-1- B3 A.albopi-1- FIP A.albopi-1- BIP A.albopi-1- LB	CCTGATATAGCTTTTCCTCGA AATAGATGAGATTCCCGCTAA TCCAGCTCCGTTTTCTACTATAGAATTTTTTTTTGAATATTACCCCCCTCT ACAGGGTGAACGGTTTATCCTTTTTTAAATCAACTGAAGCCCCAG CCTCTTTCTTCTGGAACAGCTCAT
A2	A.albopi-2-F3 A.albopi-2-B3 A.albopi-2-FIP A.albopi-2-BIP A.albopi-2-LB	GATTTGGAAACTGACTAGTACC CATGAGCTGTTCCAGAAGA AGAGGGGGGTAATATTCAAAAACTTTTTTTTAATACTAGGAGCCCCTGAT ACACTGCTGCTTTCTAGTTCTATAGTTTTAGAGGAGGATAAACCGTTCA TAGAAAACGGAGCTGGAACAGG

### LAMP reaction

The LAMP reactions were performed in a final volume of 20 μl containing the following components: 10 μl of 2×Bst 4.0 SYBR Green IsoAmp MasterMix (Anxinkang Tech. Co., LTD, Beijing, China), 16 μM of FIP and BIP, 2 μM of F3 and B3, 2 μM of LB, and 2 μl of template DNA for real-time turbidimeter. In order to prevent the aerosol contamination, the reaction system is covered with protective agent. The reactions were performed for 40 min at a constant temperature of 63°C.

The reaction was performed by a Bio-Rad iQ5 Gradient Real Time PCR system (Bio-Rad, CA, USA), and the amplification curves were extracted and analyzed. This study was repeated three times technically.

### PCR detection

PCR reactions were performed in a 25 μL reaction mixtures containing 12.5 μl PCR Master Mix reagents (Tiangen Biotech Co., Ltd, Beijing, China), 1 μM of outer forward primer (F3) and outer backward primer (B3), 1 μM of DNA template and double-distilled water. The primer sequences of F3 and B3 were obtained from the optimal primer sets in the LAMP method (**Table 1**). All primers were synthesized commercially (Sangon Biotech Co., Ltd, Shanghai, China). The amplifications conditions comprised of initial denaturation at 94°C for 5 min followed by 35 cycles of denaturation at 94°C for 30 seconds, annealing for 30 seconds, and extension at 72°C for 45 seconds. The PCR was completed with a final extension step at 72°C for 4 min. The PCR products were separated and analyzed with 1% agarose gel electrophoresis, which was stained with ethidium bromide. Then the images were documented with a Gel Doc EQ imaging system (Bio-Rad).

## Results

### Optimization of LAMP assay

**Figure 1:**
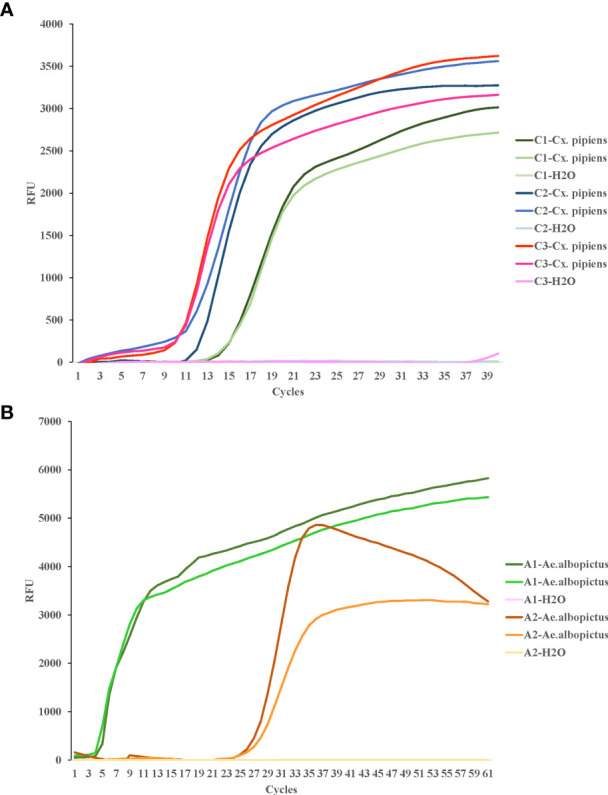
LAMP assay for the detection of target genes of *Cx. pipiens* **(A)** and *Ae. albopictus* **(B)**. C1, C2, and C3 represent the primer sets used for *Cx. pipiens* detection. A1 and A2 represent the primer sets used for *Ae. albopictus* detection. Two independent DNA templates of *Cx. pipiens* (41.4 ng/μl and 43.9 ng/μl) and *Ae. albopictus* (131.3 ng/μl and 139.5 ng/μl) were selected for amplification for each set of primers, and H_2_O was used as the negative control. The LAMP reactions were performed at a constant temperature of 65°C. This study was repeated three times technically.

**Figure 2:**
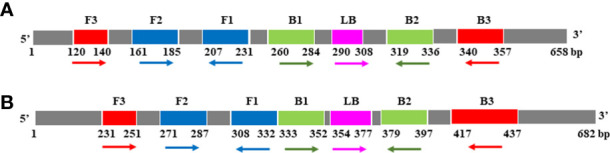
The primer design regions of the optimal primer sets for *Cx. pipiens* **(A)** and *Ae. albopictus* **(B)**. Right-pointing arrows indicate the sense sequences, left-pointing arrows indicate reverse complementary sequences.

For COI genes detection of *Cx. pipiens* and *Ae. albopictus*, three sets and two sets of primers were initially designed, respectively. The amplification reactions were carried out under the same conditions. As shown in **Figure 1**, the C3 and A1 primer sets amplified their respective target gene in the shortest time, and were therefore chosen as the optimal primer sets for LAMP detection of *Cx. pipiens* and *Ae. albopictus*, respectively. The primer design regions of the optimal primer sets were indicated in **Figure 2**.

**Figure 3:**
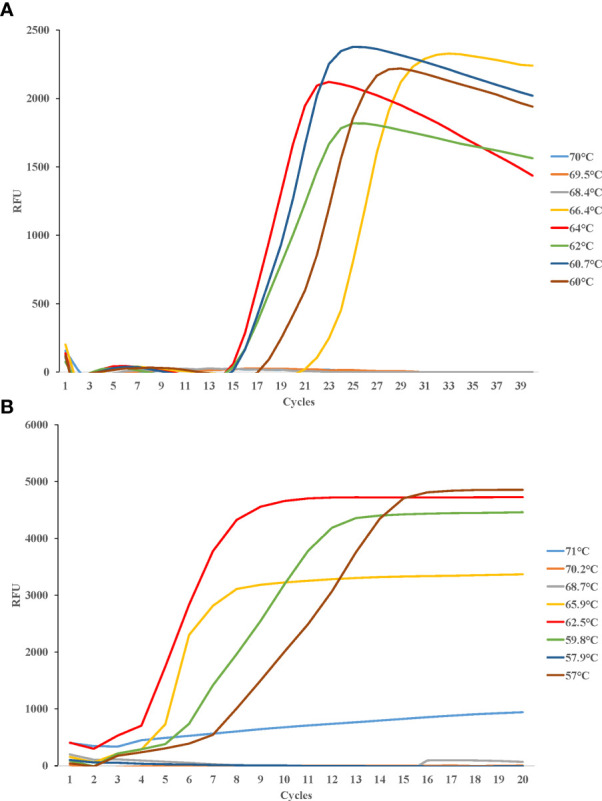
Different temperatures of LAMP reaction for detecting *Cx. pipiens* **(A)** and *Ae. albopictus* **(B)**. DNA concentrations of *Cx. pipiens* and *Ae. albopictus* were 23.7 ng/μl and 130.9 ng/μl, respectively. The reactions were performed under temperatures ranging from 60°C to 70°C for *Cx. pipiens* detection, and ranging from 57°C to 71°C for *Ae. albopictus* detection.

Eight temperature gradients were set between 60°C and 70°C for LAMP detection of *Cx. pipiens*, in the same way, eight temperature gradients were set between 57°C and 71°C for LAMP detection of *Ae. albopictus*. Fluorescence curves were compared for further optimizing the amplification. The most suitable reaction temperatures were 64°C for *Cx. pipiens*, and 62.5°C for *Ae. albopictus* (**Figure 3**).

### Specificity of LAMP detection

**Figure 4:**
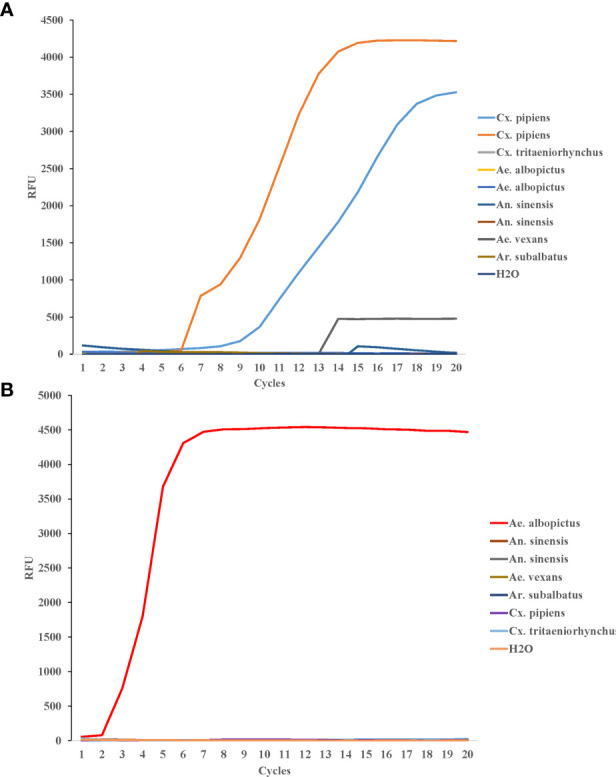
Specificity of LAMP reaction for detecting *Cx. pipiens* **(A)** and *Ae. albopictus* **(B)**. For *Cx. pipiens* detection, the DNA of two strains of *Cx. pipiens*, 1 strain of *Culex tritaeniorhynchus*, 2 strains of *Ae. albopictus*, and 2 strains of *Anopheles sinensis*, 1 strain of *Aedes vexans*, and 1 strain of *Armigeres subalbatus* were used as templates. For *Ae. albopictus* detection, the DNA of 1 strain of *Ae. albopictus*, 2 strain of *Anopheles sinensis*, 1 strain of *Ae. vexans*, 1 strain of *Ar. subalbatus*, 1 strain of *Cx. pipiens*, and 1 strain of *Cx. tritaeniorhynchus* were detected as templates. H_2_O was used as the negative control. The LAMP amplification was performed at 64°C and 62.5°C for *Cx. pipiens* and *Ae. albopictus*, respectively.

To test the LAMP specificity for COI genes of *Cx. pipiens*, The DNA of two strains of *Cx. pipiens* and seven other mosquito strains (1 strain of *Culex tritaeniorhynchus*, 2 strains of *Ae. albopictus*, and 2 strains of *Anopheles sinensis*, 1 strain of *Aedes vexans*, and 1 strain of *Armigeres subalbatus*) were used as templates. The constant temperature amplification was performed at 64°C for 20 min. The fluorescence signal was collected every 1min and the amplification curve was read for interpretation. As shown in **Figure 4A**, all *Cx. pipiens* were identified positively by LAMP assay, and the other eight strains of other mosquito species, including the blank control, were tested negative, indicating that the LAMP assay was specific for COI genes of *Cx. pipiens.*

DNA from *Ae. albopictus* and six strains of other mosquito species (*Anopheles sinensis*, *Ae. vexans*, *Ar. subalbatus*, *Cx. pipiens*, and *Cx. tritaeniorhynchus*) were detected as templates to evaluate the specificity of LAMP detection for *Ae. albopictus*. The constant temperature amplification was performed at 62.5°C for 20 min. The fluorescence signal was collected every 1min and the amplification curve was read for interpretation. The *Ae. albopictus* was identified positive by LAMP assay. The other six mosquito species and the blank control were tested negative (**Figure 4B**).

### Sensitivity of LAMP detection

**Figure 5:**
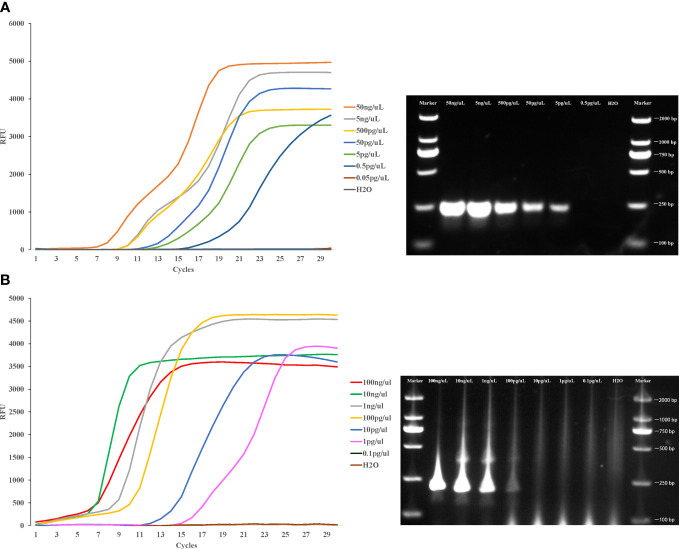
Sensitivity of the LAMP reaction in detecting *Cx. pipiens* **(A)** and *Ae. albopictus* **(B)**. Pure whole genomic DNA of *Cx. pipiens* (initial concentration was 50ng/μl) and *Ae. albopictus* (initial concentration was 100 ng/μl) strains were serially diluted 10-fold and used as templates for the LAMP and PCR assay. The LAMP amplification was performed at 64°C and 62.5°C for *Cx. pipiens* and *Ae. albopictus*, respectively. The PCR amplifications conditions comprised of initial denaturation at 94°C for 5 min followed by 35 cycles of denaturation at 94°C for 30 seconds, annealing for 30 seconds, and extension at 72°C for 45 seconds, with a final extension step at 72°C for 4 min.

To evaluate the detection limit of LAMP assay in this study, pure whole genomic DNA of *Cx. pipiens* (initial concentration was 50ng/μl) and *Ae. albopictus* (initial concentration was 100 ng/μl) strains were serially diluted 10-fold and used as template for the LAMP and PCR assay. The lower limit concentration detected by LAMP method is 0.5 pg/μl within 20 min for *Cx. pipiens*, and the sensitivity was 10 times higher than PCR method. The lower limit detected by LAMP method was 1 pg/μl within 20 min for *Ae. albopictus*, with the sensitivity 100 times higher than PCR method (**Figure 5**).

### Detection of wild mosquitoes

**Figure 6:**
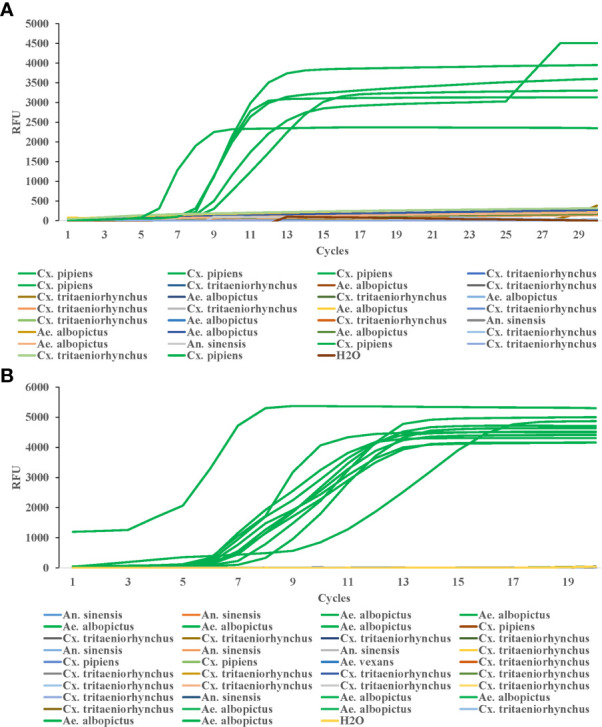
Efficiency of LAMP reaction for detecting field samples of *Cx. pipiens* **(A)** and *Ae. albopictus* **(B)**. The LAMP amplification was performed at 64°C and 62.5°C for *Cx. pipiens* and *Ae. albopictus* detection, respectively.

To test the efficiency of this method in field detection of *Cx. pipiens*, we collected 30 mosquito samples in the field, which were first identified by both traditional morphological identification and PCR, and the results showed 6 samples were *Cx. pipiens*, 9 samples were *Ae. albopictus*, 13 were *Cx. tritaeniorhynchus*, and 2 were *An. sinensis*. Then the LAMP method in this study was used to detect the DNA samples from the 30 mosquitoes, and the amplification was performed at 64°C for 30 min. The results showed that all six samples of *Cx. pipiens* showed amplification curves, while none of the other samples showed amplification curves (**Figure 6A**).

38 mosquito samples in the field were collected to test the efficiency of this method in field detection of *Ae. albopictus.* Morphological identification and RT-PCR results showed that among the 38 samples collected, 11 were *Ae. albopictus*, 6 were *An. sinensis*, 3 were *Cx. pipiens*, 17 were *Cx. tritaeniorhynchus*, and 1 was *Ae. vexans*. The LAMP method in this study was used to detect the DNA samples from the 38 mosquitoes, and the amplification was performed at 62.5°C for 20 min. The results showed that all 11 samples of *Ae. albopictus* showed amplification curves, while none of the other samples showed amplification curves (**Figure 6B**).

There is a slight difference (about 2 minutes) in the time of peak onset, which may be related to the initial concentration of the samples and the technical deviation.

## Discussion

*Cx. pipiens* and *Ae. albopictus* are closely related to human life, and carry and transmit a variety of viruses, causing serious harm to human health (14, 15). At present, species identification of mosquitoes mostly relies on morphological identification. However, morphological identification methods are limited by many factors, such as damaged specimens, similar morphology of close-related species, and difficult identification of imported species (16). More importantly, new mosquito species could become vectors of new diseases, and monitoring of mosquito species in a geographical area is crucial (17). The LAMP detection method in this study solved this problem, providing a rapid detection method for the identification of *Cx. pipiens* and *Ae. albopictus*. The advantage of LAMP in sensitivity has been reported in many studies (18, 19). Our study showed significant advantages especially for rapid and accurate identification of large number of samples in the field.

For rapid and accurate identification of large number of samples, LAMP has irreplaceable advantages. Firstly, the LAMP assay does not require complex instruments and procedures (20). In comparison with PCR assay, which needs complex temperature-cycling conditions, the LAMP assay only needs a constant temperature environment, is simple and easy to achieve, which providing a great convenience for the clinical and on-site rapid detection (20). Secondly, the LAMP assay has low requirement for the sample’s purity, which saves time for the detection of large number of samples on-site (18). The purification of DNA from a complex sample was not necessary as described by Kaneko who suggested that the LAMP reaction was not susceptible to the influence of the impurities often presented in samples (13). Even if the captured mosquito is mutilated or destroyed, we can still use LAMP to accurately identify the species.

In conclusion, LAMP detection method has dominant advantages (18–21), including rapidity, easy operability, high sensitivity and specificity, etc., and has significant advantages in rapid detection of large number of samples (21). In this study, we established a novel LAMP method based on COI gene, which was rapid, sensitive, specific, and effective for the *Cx. pipiens* and *Ae. albopictus* detection, and this method was expected to be used for species identification of a large number of samples in the field. However, the higher sensitivity of the LAMP method is also a double-edged sword, and false positives are relatively common in LAMP reactions. This is due to the diffusion of aerosols formed by LAMP products into the laboratory environment and the contamination of lab coats, pipettes, reagents, materials, equipment, etc. Therefore, to ensure the accuracy of the experiment, the LAMP reaction needs to be performed in an independent laboratory with a good molecular biology foundation. In the future, multiple LAMP methods for mosquito species identification and arbovirus screening will have long-term research significance and value.

## Data availability statement

The original contributions presented in the study are included in the article/supplementary material. Further inquiries can be directed to the corresponding author.

## Author contributions

XW designed research; BM and YL performed research; HP and XZ contributed new reagents or analytic tools; XW and XZ analyzed data; XZ wrote the paper. All authors contributed to the article and approved the submitted version.
